# Epidemiology of Paediatric Italian Food Allergy: Results of the EPIFA study

**DOI:** 10.1016/j.jacig.2024.100246

**Published:** 2024-03-26

**Authors:** Rita Nocerino, Laura Carucci, Serena Coppola, Gaetano Cecere, Maria Micillo, Tina Castaldo, Stefania Russo, Marialuisa Sandomenico, Antonio Marino, Renato Gualano, Paola Ercolini, Antonella Capasso, Giorgio Bedogni, Roberto Berni Canani, Italian Society of Pediatric Gastroenterology and Nutrition (SIGENP)

**Affiliations:** a Department of Translational Medical Science, University of Naples Federico II, Naples, Italy; b ImmunoNutritionLab at CEINGE Advanced Biotechnologies, University of Naples Federico II, Naples, Italy; c European Laboratory for the Investigation of Food-Induced Diseases, University of Naples Federico II, Naples, Italy; d Task Force for Microbiome Studies, University of Naples Federico II, Naples, Italy; e Department of Medical and Surgical Sciences, Alma Mater Studiorum University of Bologna, Bologna, Italy; f Department of Internal Medicine, S Maria delle Croci Hospital, AUSL Romagna, Ravenna, Italy

**Keywords:** Prevalence, incidence, cow’s milk allergy, hen’s egg allergy, nut allergy, anaphylaxis, food allergens, children, oral food challenge

## Abstract

**Background:**

Updated epidemiologic data are important for defining effective public health strategies for pediatric food allergy (FA).

**Objective:**

The Epidemiology of Paediatric Italian Food Allergy (EPIFA) study was designed to investigate the epidemiology of pediatric FA in one of the most heavily populated Italian regions.

**Methods:**

A retrospective cohort study was performed in collaboration with family pediatricians aimed at investigating the epidemiology of Italian pediatric FA during 2009 to 2021. Family pediatricians in the Campania region were invited to use the Google Forms platform for online compilation of data forms. Data forms were reviewed by experienced pediatric allergists at the coordinating center.

**Results:**

A total population of 105,151 subjects (aged 0-14 years) was screened during the study period. Data from 752 FA patients were evaluated. A progressive increase in FA incidence and prevalence was observed from 2009 to 2021, with a relative increase up to 34% and 113.6%, respectively, at the end of study period. The relative increase in FA prevalence was higher in the 0-3-year-old age group in the same study period (+120.8%). The most frequent allergens were cow’s milk, hen’s egg, and nuts.

**Conclusion:**

The results of the EPIFA study showed an increase in pediatric FA incidence and prevalence from 2009 to 2021 in Italy. These results underline the necessity of new effective strategies for preventing and managing these conditions.

Food allergy (FA) is a significant global public health problem in the pediatric age.^1^, ^2^, ^3^, ^4^, ^5^, ^6^ Several studies have suggested that the prevalence of pediatric FA is increasing worldwide.^7^, ^8^, ^9^, ^10^, ^11^, ^12^ Population-based studies on the epidemiology of the Italian pediatric population in recent decades are scarce.^13^ Available data derive mainly from self-reported diagnosis, which results in an overall risk of bias.^14^^,^^15^ Data on the Italian population included in the EuroPrevall trial were limited to a small number of subjects. Despite the initial inclusion of Italy in the EuroPrevall study, subsequent investigations have not reported data from the Italian pediatric population.^5^^,^^14^^,^^16^, ^17^, ^18^, ^19^

FA is also known to be the main cause of anaphylaxis in pediatric patients presenting to hospital emergency departments.^20^, ^21^, ^22^ A significant increase in the number of children requiring hospitalization for food-induced anaphylaxis has been observed in Italy from 2001 to 2011.^23^^,^^24^ This pattern is in line with data reported in other countries.^25^, ^26^, ^27^, ^28^, ^29^

The prevalence and trend of food hypersensitivity in the community depend on public awareness, diagnostic methods, and dietary habits of the population, and could vary between geographical regions.^30^^,^^31^ Updated epidemiologic data on pediatric FA are important not only to improve the awareness of FA but also to facilitate the development of new effective public health strategies for the prevention and management of these conditions.

The Epidemiology of Paediatric Italian Food Allergy (EPIFA) project was launched to investigate the current epidemiologic scenario of pediatric FA in Italy.

## Methods

### Study design

The EPIFA project was a retrospective cohort study promoted by the Italian Society of Paediatric Gastroenterology and Nutrition. The study was performed in collaboration with family pediatricians (FPs), who care for children up to 14 years of age in the Italian public health system. The aim of the project was to investigate the epidemiology of pediatric FA in the years 2009-2021. The study was performed in the Campania region, one of the most populous Italian regions (75,094,559 cumulative inhabitants from 2009 to 2021), with a total pediatric population of 11,617,196 subjects (age range 0-14 years from 2009 to 2021).^32^

### Data collection and analysis

The EPIFA project was designed and coordinated by the clinical research team of the Paediatric Allergy Program of the Department of Translational Medical Science of the University of Naples Federico II. The clinical research team was composed of pediatricians, pediatric allergists, dietitians, allergy nurses, and biostatisticians.

**Fig 1:**
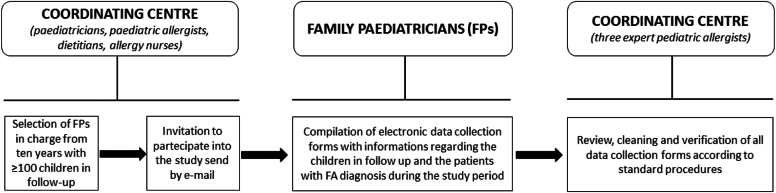
Study design.

The study’s design is depicted in Fig 1. Using the public register of the FPs operating in the Campania region, the clinical research team randomly selected 4 FPs from each of the 5 provinces of the Campania region that had been in charge for at least 10 years and had an annual cohort of at least 700 children. The FPs received a formal invitation by email. The FPs, who agreed to participate in the study, were then invited to one kickoff teleconference to receive information about the study design. After this kickoff teleconference, each FP was also provided with written instructions on the data collection method and the contact details of the research assistants, who could be contacted in real time in case of doubts or questions.

The FPs were requested to provide information on the annual number of children followed up and the number of patients with a diagnosis of FA during the years 2009-2021. Then, for each patient with FA, main demographic and clinical information such as sex, presence of allergy family risk, age at FA diagnosis, FA symptoms, responsible food allergens, adopted diagnostic assessment, and age at tolerance acquisition were collected. Each patient was identified with a unique ID, initials of first name and surname, place of birth, and Italian tax identification number, thus avoiding the same patient’s being included 2 or more times. The FPs were invited to use the Google Forms platform for the online compilation of the data collection form. All data collection forms provided by the FPs were evaluated by the clinical research team at the coordinating center. The investigators reviewed the data collection forms for completeness, clarity, consistency, and accuracy, and assessed the diagnostic work-up adopted for each FA case as follows. Random and independent backchecks were also performed. All FA cases reported by the FPs in which the data collection forms were incomplete or reported no suggestive clinical features or an unclear response to the elimination diet were excluded from analysis.

Two diagnostic scenarios were considered, as previously adopted by others:^33^ (1) convincing FA, which was based on the occurrence of suggestive clinical features of FA (urticaria, angioedema, atopic dermatitis, asthma, oculorhinitis, diarrhea, regurgitation/vomiting, abdominal pain, constipation, bloody stools, failure to thrive) after the ingestion of a specific food and a clear response to the elimination diet; and (2) confirmed FA, which was based on the presence of clinical features suggestive of FA, clear response to the elimination diet, positive FA screening test results (skin prick test [SPT], serum-specific IgE, atopy patch test), and/or positive oral food challenge (OFC). The acquisition of immune tolerance was evaluated by the negative outcome of the OFC to the trigger allergen.

**Fig 2:**
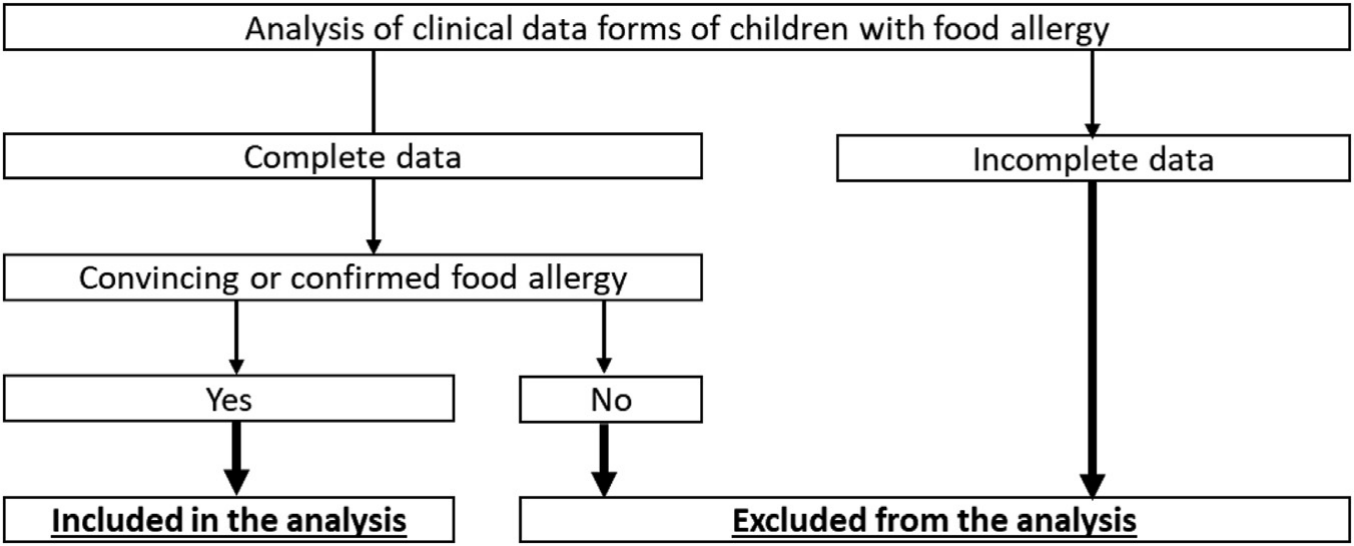
Study procedures. Two diagnostic scenarios were considered: (1) convincing FA diagnosis, based on occurrence of suggestive clinical features of FA (urticaria, angioedema, atopic dermatitis, asthma, oculorhinitis, diarrhea, regurgitation/vomiting, abdominal pain, constipation, bloody stool, failure to thrive) after ingestion of specific food and clear response to elimination diet; and (2) confirmed FA diagnosis, based on presence of clinical features suggestive of FA, clear response to elimination diet, positive FA screening test results (SPT, serum specific IgE test, atopy patch test), and/or positive OFC.

Study procedures are summarized in Fig 2.

### Statistical analysis

Using a single data-entry method, all recorded data were entered in the study database. Then the study data set was reviewed and underwent data cleaning and verification according to standard procedures. Finally, the database was locked once it was declared complete and accurate.

The Kolmogorov-Smirnov test was used to determine whether variables were normally distributed. Descriptive statistics were reported as the means and standard deviations for continuous variables, and discrete variables were reported as the number and proportion of subjects with the characteristic of interest.

Prevalence was defined as the number of current cases divided by the entire population during a given year. Incidence was likewise defined as the number of new cases divided by the entire population during a given year. Using tabular data, we quantified the year-related changes in prevalence and incidence using a binomial regression model with prevalence or incidence as outcome (0 = event absent; 1 = event present), the total population as (frequency) weight, and discrete year as predictor. We evaluated the change in prevalence and incidence by contrasting each year (2010 to 2021) with the starting year (2009). Because 12 contrasts were involved, we performed a Bonferroni correction for 12 comparisons and report corrected 95% confidence interval and *P* values. Considering the FA prevalence value in 2021, we performed an estimation of FA prevalence in the general pediatric (age 0-14 years) Italian population in the same year.

Statistical analysis was performed by a statistician using SPSS for Windows 23.0 (IBM, Armonk, NY) and Stata 17.0 (StataCorp, College Station, Tex).

### Ethical approval

The study was approved by the ethics committee of University Federico II of Naples and was performed in accordance with the Helsinki Declaration (Fortaleza revision, 2004) and according to good clinical practice standards (CPMP/ICH/135/95), and with the pertinent European and Italian privacy regulations. Written informed consent was obtained from the parents or caregivers of each subject.

## Results

### Study population

**Fig 3:**
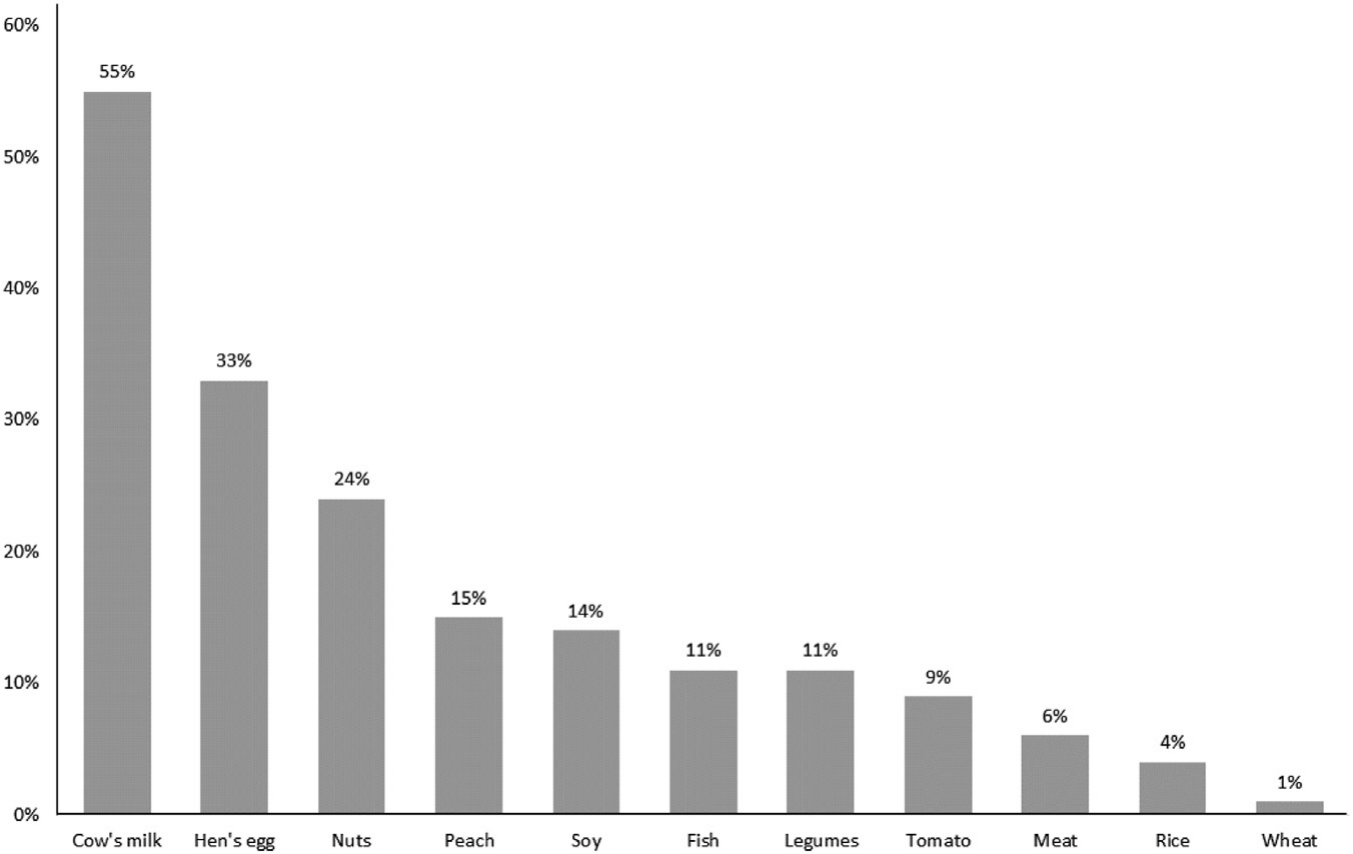
Most frequent food allergens responsible for pediatric FA in study population.

Ten of 20 FPs who received the study invitation agreed to participate in the EPIFA study. The FPs were 2 from each province of the Campania region and had a total population of 105,151 subjects aged 0-14 years during the study period. The FPs provided clinical data forms of 854 suspected FA patients followed from 2009 to 2021. The data forms were analyzed by the clinical research team; 25 forms were considered ineligible because data were missing, and 77 forms were considered inconclusive for FA diagnosis, all due to an unclear response to the elimination diet and/or the OFC. Thus, clinical data forms from 752 children with FA were entered into the electronic database for analysis. Main data on medical history (ie, mode of delivery; gestational age; birth weight, length, and head circumference; breast-feeding; weaning age; family history of allergies) and demographic characteristics of the study population are reported in Table I. The majority of patients (56.9%) were male, and more than 60% of the subjects were born by caesarean section and had a familial allergy risk, with at least one first-degree allergic family member (57.7%). The main clinical features of children with FA evaluated in the study are reported in Table II. More than 55% of the patients presented with multiple FA; most presented with gastrointestinal symptoms. The most frequent allergens responsible for FA were cow’s milk proteins and hen’s egg (Fig 3).

**Table I:** Characteristics of study population

Characteristic	Value
No. of children with FA	752
Familial allergy risk	496 (65.9)
No. of allergic relatives	
1	286 (57.7)
2	179 (36.1)
≥3	31 (6.2)
Familiarity for FA	196 (26.1)
Male sex	428 (56.9)
Urban setting	405 (53.8)
Cesarean delivery	462 (61.4)
Born at term	682 (90.7)
Birth weight (g)	3156 ± 650
Birth length (cm)	49.4 ± 2.8
Birth head circumference (cm)	35.3 ± 1.7
Exclusive breast-feeding for at least 4 months	253 (33.6)
Duration (months) of breast-feeding	6.9 ± 4.5
Age (months) at weaning	5 ± 0.9

Data are presented as nos. (%) or means ± standard deviations unless otherwise indicated.

**Table II:** Clinical features of study population

Characteristic	Value
IgE-mediated FA mechanism	535/752 (71.1)
Age (months) at symptom onset, median (IQR)	12 (61)
Multiple FAs	419 (55.7)
Positive SPT result in subjects with confirmed FA diagnosis	535/640 (83.6)
Positive atopy patch test in subjects with confirmed FA diagnosis	105/640 (16.4)
Subjects with OFC to confirm FA diagnosis	270/640 (42.2)
Symptoms	
Cutaneous	290 (38.6)
Only cutaneous	173 (59.6)
Cutaneous + respiratory	75 (25.9)
Cutaneous + gastrointestinal	42 (14.5)
Gastrointestinal	345 (45.9)
Only gastrointestinal	220 (63.8)
Gastrointestinal + cutaneous	107 (31)
Gastrointestinal + respiratory	18 (5.2)
Respiratory	128 (17)
Only respiratory	31 (24.2)
Respiratory + gastrointestinal	20 (15.6)
Respiratory + cutaneous	77 (60.1)
Anaphylaxis	198 (26.3)
Patients outgrown FA	390 (51.9)
IgE mediated	159/390 (40.8)
Non-IgE mediated	231/390 (59.2)
Age (months) at immune tolerance acquisition	45.7 ± 28.4

Data are presented as nos. (%), means ± standard deviations, or n/N (%) unless otherwise indicated.

The diagnosis of FA was made in 397 subjects (52.8%) at a tertiary-care center for pediatric allergy, for 248 (33%) at a secondary pediatric care center, and in 107 (14.2%) at offices of FPs. Using the FA scenarios described above, we found a FA diagnosis convincing in 112 cases (14.9%) and confirmed in 640 cases (85.1%). All 640 confirmed FA cases had at least 1 positive FA screening test (83.6% positive SPT, while 16.4% positive atopy patch test), and in 270 cases (42.2%), FA diagnosis was confirmed by OFC. The frequency of FA diagnoses was similar among the different FPs involved in the study.

During the study period, the acquisition of immune tolerance was reported in 390 children (51.9%), of whom 323 were within 5 years of age (82.8%), 72 within 10 years of age (18.5%), and 5 within 14 years of age (1.3%).

### Epidemiology of pediatric FA

**Fig 4:**
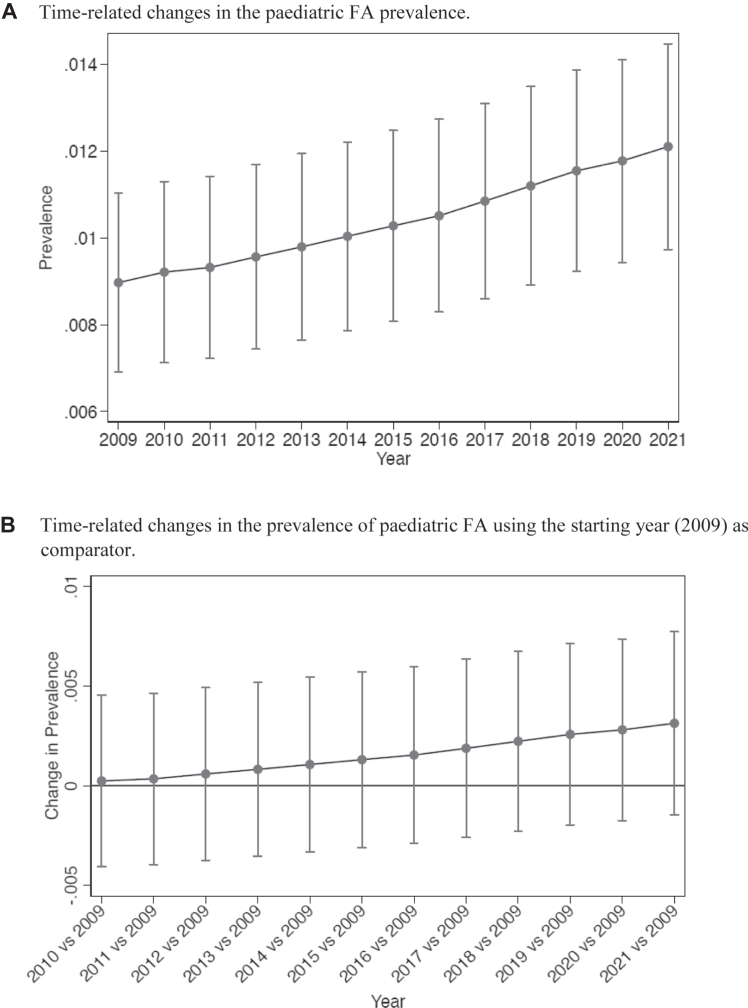
**(A)** Time-related changes in incidence of disease. Values are means and 95% confidence intervals (CIs) from binomial regression. **(B)** Time-related changes in incidence of disease using starting year (2009) as comparator. Values are means and 95% CIs from binomial regression with Bonferroni correction for 12 comparisons. Values whose 95% CIs do not cross horizontal line are statistically significant at *P* < .05.

**Fig 5:**
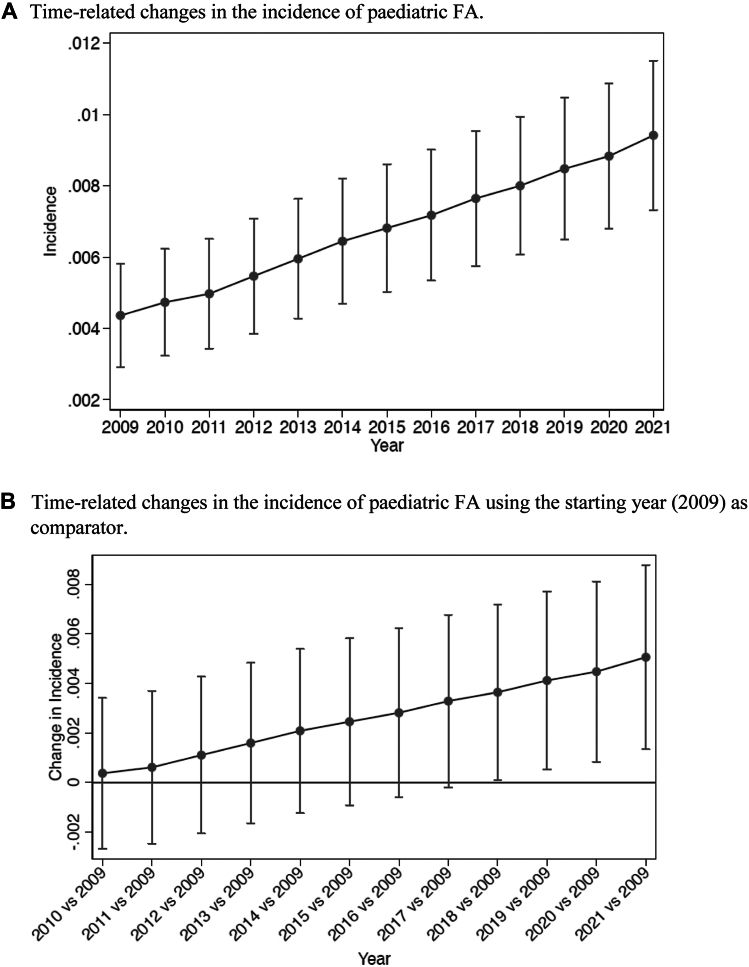
**(A)** Time-related changes in prevalence of disease. Values are means and 95% confidence intervals (CIs) from binomial regression. **(B)** Time-related changes in prevalence of disease using starting year (2009) as comparator. Values are means and 95% CIs from binomial regression with Bonferroni correction for 12 comparisons. Values whose 95% CIs do not cross horizontal line are statistically significant at *P* < .05.

A progressive increase in pediatric FA prevalence (from 0.009 to 0.0121, a relative increase of 34.4%) (Fig 4) and incidence (from 0.0044 to 0.0094, a relative increase of 113.6%) (Fig 5) was observed from 2009 to 2021. The binomial regression model revealed a significant change in FA incidence from the year 2018 compared to the starting year (2009).

**Fig 6:**
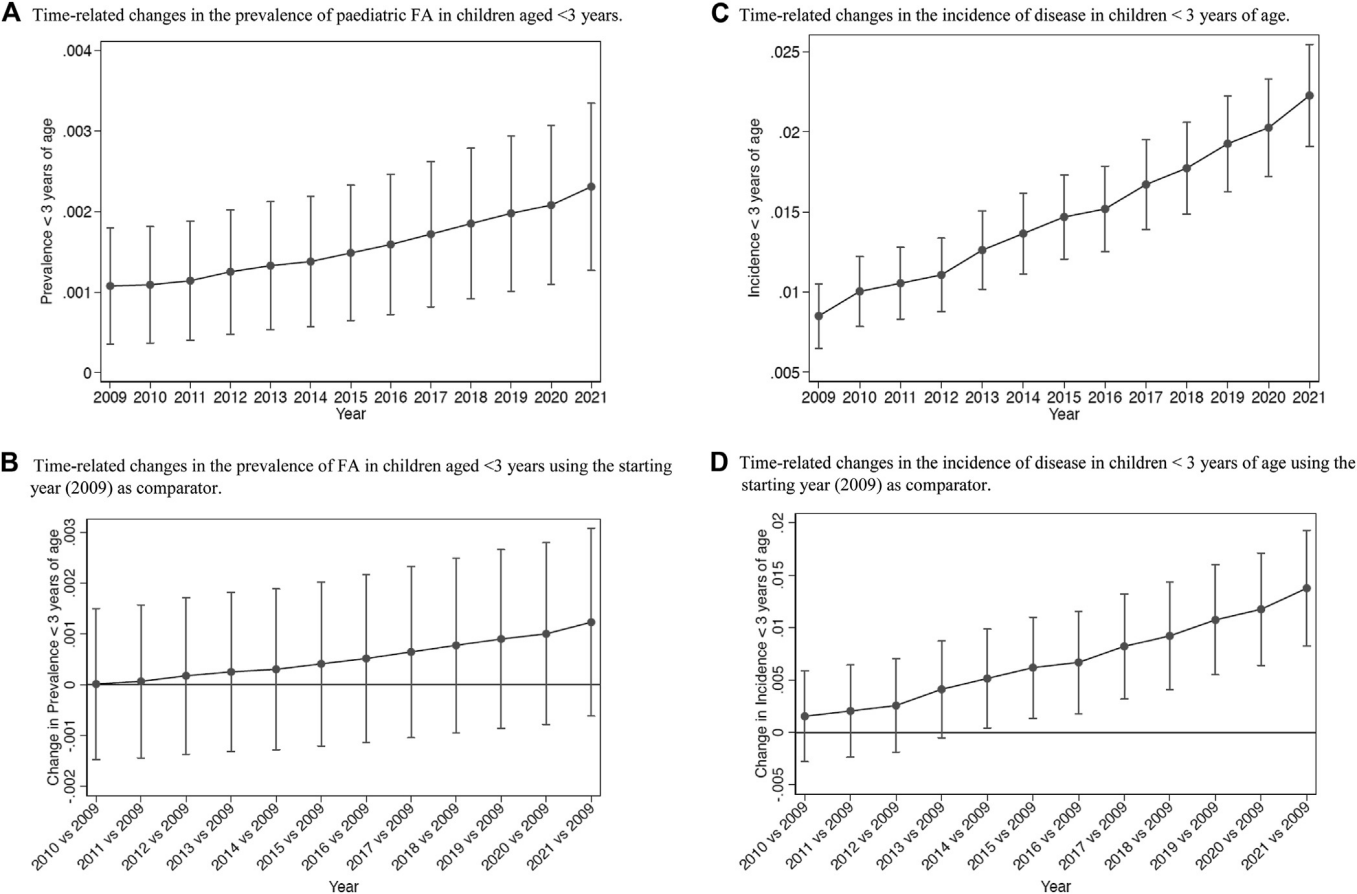
**(A)** Time-related changes in incidence of disease in children <3 years of age. Values are means and 95% confidence intervals (CIs) from binomial regression. **(B)** Time-related changes in incidence of disease in children <3 years of age using starting year (2009) as comparator. Values are means and 95% CIs from binomial regression with Bonferroni correction for 12 comparisons. Values whose 95% CIs do not cross horizontal line are statistically significant at *P* < .05. **(C)** Time-related changes in prevalence of disease in children <3 years of age. Values are means and 95% CIs from binomial regression. **(D)** Time-related changes in prevalence of disease in children aged <3 years using starting year 2009 as comparator. Values are means and 95% CIs from binomial regression with Bonferroni correction for 12 comparisons. Values whose 95% CIs do not cross horizontal line are statistically significant at *P* < .05.

The increase in FA prevalence (from 0.0202 to 0.0446, a relative increase of 120.8%) and incidence (0.0085 to 0.0223, a relative increase of 162.3%) during the study period was higher in children aged ≤3 years (Fig 6). Also in this case, the binomial regression model revealed a significant change in FA incidence from the year 2014 compared to the starting year (2009).

Extending these findings to the general pediatric (0-14 years) Italian population totaling 7,636,545 subjects in the year 2021,^32^ we estimated a total number of 92,445 children with FA in Italy in that same year.

## Discussion

FA is one of the most common allergic conditions in pediatric age.^34^ Detailed and updated epidemiologic data are important to better define effective strategies for FA prevention and treatment. The EPIFA study was the first investigation on epidemiologic features of pediatric FA from 2009 to 2021 in Italy. The study was conducted in one of the most populated Italian regions. The Campania region is the third most populated region in Italy and the most densely populated, with an area of 13.590 km^2^.^32^ The study population could be considered well representative of the general Italian pediatric population, with similar exposure to major environmental factors compared to children living in other Italian regions, and with a socioeconomic status that is well representative of the majority of Italian families.^32^ In line with what has recently been reported in other countries,^1^, ^2^, ^3^, ^4^, ^5^, ^6^ we observed a progressive increase in FA incidence and prevalence in the Italian pediatric population over the last few years. The prevalence of pediatric FA observed in the EPIFA study was in line with the most recent estimates of the prevalence and trends of FA in Europe.^7^ Our results differ from those reported in EuroPrevall study, supporting their conclusion that large geographical differences in the prevalence of FA in school-age children could be observed across Europe.^5^ These differences may be due to environmental and climatic differences; they may also reflect the level of public awareness of FA in each country. In addition, recently updated estimates of FA have shown that there are no consistent patterns across European regions, probably because of heterogeneities within and between European regions and age groups as well as methodologic differences—for example, in the definition of FA across Europe.^31^

Although OFC is considered the reference standard for FA diagnosis, it was not considered as an inclusion criterion in the EPIFA study, as was also adopted in other studies.^33^^,^^35^, ^36^, ^37^, ^38^ It should be highlighted that up to 52.8% of patients were diagnosed in a tertiary-care center for pediatric allergy and that in up to 85.1% of cases, the diagnosis was considered confirmed by the results of FA screening tests and/or positive OFC. Finally, 26.3% of patients presented with a history of anaphylaxis. In these patients, when a single responsible food (not a composite meal eaten) was involved, the OFC was considered unnecessary to obtain a FA diagnosis.^39^

In addition to data on FA incidence and prevalence over the 13-year period of observation, the EPIFA study also provided information on the clinical features and natural history of pediatric FA. As reported also by others,^12^^,^^40^ the influence of genetic background is relevant for the occurrence of FA, as suggested by the higher prevalence of FA in male subjects with a positive family risk for atopy. Similarly, the most common clinical manifestations of FA involve the skin and the gastrointestinal tract, as previously reported by others.^41^

Cow’s milk protein allergy was confirmed as the most common form of FA in the Italian pediatric population as well.^42^^,^^43^ The other most common food allergens reported in EPIFA study—nuts, peach, and soy—could suggest an increase in allergy to lipid transfer protein, as reported in other countries.^44^^,^^45^

Results on the natural history of pediatric FA confirm data observed in other countries.^40^^,^^46^ Immune tolerance was acquired in only 51.9% of cases, suggesting a modification of FA’s natural history with a lower rate of acquisition of immune tolerance and the need for longer follow-up for these conditions. These data also suggest a negative impact resulting from costs related to FA management for both families and health care systems.^47^

Major limitations of this study could derive from the evaluation of only a single Italian region and from the relatively small number of FPs reporting FA cases in children. In addition, the rate of FPs who agreed to participate in the study was 50%; this could have led to potential nonresponse bias or to possible selection bias, with physicians more interested in FA more likely to participate in the study. In addition, several patients with FA were diagnosed only in the last years of the study period, resulting in a shorter follow-up period; this could make difficult to collect data on the clinical outcomes of these patients. The major strengths of this study are related to the fact that only diagnoses made by physicians were included, and that all reported cases by the FPs were reviewed to exclude cases in which the FA diagnosis was inconsistent. Last, we report data on the epidemiologic temporal trends and natural history of pediatric FA in a large, well-defined cohort of subjects.

In conclusion, the results of the EPIFA study suggest an increase in the incidence and prevalence of pediatric FA in Italy from 2009 to 2021. These data underline the need for new and effective strategies for the prevention and the management of these conditions, together with more efficient supportive policies and initiatives for the families of these patients.

## Disclosure statement

This study was supported by the Department of Translational Medical Science of the University of Naples “Federico II,” Naples, Italy, which received funding from the National Recovery and Resilience Plan, European Union–Next Generation EU (On Foods–Research and Innovation Network on Food and Nutrition Sustainability, Safety and Security—Working on Foods; code PE0000003) and from the Italian Ministry of Health - Health Operational Plan Trajectory 5 - Line of action “Creation of an action program for the fight against malnutrition in all its forms and for the dissemination of the principles of the diet Mediterranean” (Mediterranean Diet for Human Health Lab “MeDiHealthLab”; code T5-AN-07). The funder had no influence on study design; collection, analysis, and interpretation of data; the writing of the report; and the decision to submit the report for publication.

Disclosure of potential conflict of interest: The authors declare that they have no relevant conflicts of interest.

**Key messages**

• Data on the epidemiology of FA in the Italian pediatric population are scarce.
• The study is the first Italian estimate on pediatric FA (2009-2021) based only on sure diagnoses.
